# Sex Differences in Characteristics and Outcomes Among Patients With Hypertrophic Cardiomyopathy: Insights From the REVEAL‐HCM Study

**DOI:** 10.1161/JAHA.125.046708

**Published:** 2026-06-23

**Authors:** Masayuki Nakamura, Haruhiko Abe, Yasunori Ueda, Hiroaki Kitaoka, Yasushi Sakata, Kaoru Dohi, Yukichi Tokita, Takao Kato, Shouji Matsushima, Masashi Amano, Takeshi Kitai, Atsushi Okada, Yutaka Furukawa, Toshihiro Tamura, Akihiro Hayashida, Kenji Ando, Satoshi Yuda, Moriaki Inoko, Kazushige Kadota, Yukio Abe, Katsuomi Iwakura, Tetsuya Kitamura, Jun Masuda, Takahiro Ohara, Takashi Omura, Takashi Tanigawa, Kenji Nakamura, Kiyomasa Nakatsuka, Kunihiro Nishimura, Chisato Izumi, the REVEAL‐HCM Investigators

**Affiliations:** ^1^ Cardiovascular Division NHO Osaka National Hospital Osaka Japan; ^2^ Department of Cardiology and Geriatrics, Kochi Medical School Kochi University Kochi Japan; ^3^ Department of Cardiovascular Medicine Osaka University Graduate School of Medicine Osaka Japan; ^4^ Department of Cardiology and Nephrology Mie University Graduate School of Medicine Tsu Japan; ^5^ Department of Cardiovascular Medicine Nippon Medical School Tokyo Japan; ^6^ Department of Cardiovascular Medicine, Faculty of Medicine Kyoto University Graduate School of Medicine Kyoto Japan; ^7^ Department of Cardiovascular Medicine, Faculty of Medical Sciences Kyushu University Fukuoka Japan; ^8^ Department of Heart Failure and Transplantation National Cerebral and Cardiovascular Center Suita Japan; ^9^ Department of Cardiovascular Medicine Kobe City Medical Center General Hospital Kobe Japan; ^10^ Department of Cardiology Tenri Hospital Tenri Japan; ^11^ Department of Cardiology The Sakakibara Heart Institute of Okayama Okayama Japan; ^12^ Department of Cardiology Kokura Memorial Hospital Kitakyushu Japan; ^13^ Department of Cardiology Teine Keijinkai Hospital Sapporo Japan; ^14^ Department of Cardiovascular Center Medical Research Institute Kitano Hospital Osaka Japan; ^15^ Department of Cardiovascular Medicine Kurashiki Central Hospital Kurashiki Japan; ^16^ Department of Cardiology Osaka City General Hospital Osaka Japan; ^17^ Department of Cardiovascular Center Sakurabashi Watanabe Hospital Osaka Japan; ^18^ Department of Cardiology Suzuka General Hospital Suzuka Japan; ^19^ Department of Cardiology Mie Prefectural General Medical Center Yokkaichi Japan; ^20^ Division of Geriatric and Community Medicine Tohoku Medical and Pharmaceutical University Sendai Japan; ^21^ Department of Cardiology Kuwana City Medical Center Kuwana Japan; ^22^ Department of Cardiology Matsusaka Chuo General Hospital Matsusaka Japan; ^23^ Department of Cardiology Ise Red Cross Hospital Ise Japan; ^24^ Department of Preventive Medicine and Epidemiology National Cerebral and Cardiovascular Center Suita Japan

**Keywords:** hypertrophic cardiomyopathy, outcomes, sex difference, Clinical Studies, Cardiovascular Disease, Women, Cardiomyopathy, Mortality/Survival

## Abstract

**Background:**

Female sex has been associated with poor prognosis in hypertrophic cardiomyopathy (HCM), but the factors contributing to this disparity remain insufficiently defined. We aimed to clarify sex differences in clinical characteristics and factors associated with cardiovascular death in Japan.

**Methods:**

This multicenter, retrospective observational study of HCM was conducted between January 1, 2006, and December 31, 2018 (REVEAL‐HCM study [Multicenter Registry to Evaluate Risk Factors for Disease Progression, Sudden Cardiac Death and Adverse Clinical Outcomes in Japanese Patients With Hypertrophic Cardiomyopathy]; UMIN000046932). Patients aged ≥16 years with HCM were enrolled. Baseline characteristics and clinical outcomes including cardiovascular death were assessed. Univariable and multivariable Cox proportional hazards models were used to identify factors associated with cardiovascular death.

**Results:**

Of 3247 patients (median age, 67 years; 43% women), women were older and more symptomatic at presentation (52% New York Heart Association class II–IV versus 35% in men). Cardiovascular death was more common in women (hazard ratio [HR], 1.37 [95% CI, 1.02–1.82]; *P*=0.03). In multivariable analysis, older age (per 1‐year increase; HR, 1.04 [95% CI, 1.02–1.06]), advanced New York Heart Association class (HR, 1.99 [95% CI, 1.40–2.82]), history of atrial fibrillation (HR, 1.55 [95% CI, 1.10–2.18]), greater maximal wall thickness indexed to body surface area (per 1‐mm/m^2^ increase; HR, 1.12 [95% CI, 1.06–1.20]), and apical HCM (HR, 0.53 [95% CI, 0.32–0.88]) were independently associated with cardiovascular death, attenuating the HR for female sex.

**Conclusions:**

Sex differences in clinical characteristics and outcomes were observed. The excess cardiovascular death in women with HCM was largely explained by older age, more advanced symptoms, greater indexed wall thickness, and sex‐specific differences in both the prevalence and prognostic impact of apical HCM.

**Nonstandard Abbreviations and Acronyms**
AADs: antiarrhythmic drugs
ES‐HCM: end‐stage hypertrophic cardiomyopathy
HCM: hypertrophic cardiomyopathy
HOCM: hypertrophic obstructive cardiomyopathy
MWT: maximal wall thickness
NYHA: New York Heart Association
REVEAL‐HCM: Multicenter Registry to Evaluate Risk Factors for Disease Progression, Sudden Cardiac Death and Adverse Clinical Outcomes in Japanese Patients With Hypertrophic Cardiomyopathy
SCD: sudden cardiac death

**Clinical Perspective**

**What Is New?**

- This study systematically evaluated multiple previously reported factors and identified those associated with adverse outcomes in female patients.
- Inappropriate diagnostic criteria, underdiagnosis in women, and differences in morphological phenotype were associated with worse clinical outcomes in female patients with hypertrophic cardiomyopathy.

**What Are the Clinical Implications?**

- Our findings suggest that improving access to health screening and refining diagnostic criteria, particularly with consideration of sex‐specific differences, may contribute to improved outcomes in women with hypertrophic cardiomyopathy.

Hypertrophic cardiomyopathy (HCM) is an inherited myocardial disorder characterized by substantial heterogeneity in morphologic and clinical phenotypes.^1^, ^2^ Variations in the extent and distribution of left ventricular (LV) hypertrophy, the presence or absence of LV outflow tract obstruction, and the severity of diastolic dysfunction contribute to a broad spectrum of clinical outcomes, ranging from sudden cardiac death (SCD) and progressive heart failure to an asymptomatic course.

Sex differences have been recognized as important modifiers of disease expression and prognosis in coronary artery disease, heart failure, valvular heart disease, and atrial fibrillation (AF).^3^, ^4^, ^5^, ^6^ Sex differences have also been reported in HCM, with most studies showing poor outcomes in women.^7^, ^8^, ^9^, ^10^, ^11^, ^12^, ^13^, ^14^, ^15^ Potential explanations include delayed diagnosis in women, diagnostic criteria that do not adjust for body size, sex bias in medical management, and differences in the morphological subtypes of HCM.^9^, ^16^, ^17^, ^18^ Because multiple factors contribute to sex differences in HCM, a comprehensive and systematic evaluation that incorporates these factors is warranted; however, such studies remain limited.

The present study aimed to identify sex differences in clinical characteristics and to determine the clinical and morphological factors associated with the poor prognosis observed in female patients with HCM, using data from a large‐scale Japanese observational registry.

## Methods

### Data Availability

The data that support the findings of this study are available from the corresponding author upon reasonable request.

### Study Design and Population

This multicenter, retrospective observational study included patients with HCM and end‐stage HCM (ES‐HCM) from 23 tertiary care hospitals in Japan between January 2006 and December 2018 (REVEAL‐HCM [Multicenter Registry to Evaluate Risk Factors for Disease Progression, Sudden Cardiac Death and Adverse Clinical Outcomes in Japanese Patients With Hypertrophic Cardiomyopathy]; UMIN000046932).^19^

The enrollment date for each patient was defined as the date corresponding to the earliest echocardiographic examination recorded in the medical records. On the enrollment date, baseline clinical information, including demographic characteristics, medical history, and imaging findings, was collected. Longitudinal prognostic data were subsequently obtained by tracing medical records from the enrollment date through the most recent follow‐up available. Patients eligible for this study were required to be aged at least 16 years at the time of enrollment. All participants had been diagnosed with HCM either before or at the time of enrollment. Accordingly, for some patients, the date of enrollment may have differed from the actual date of HCM diagnosis, reflecting variations in the timing of their clinical evaluation and data collection. This potential time gap was carefully considered in the analysis of clinical outcomes. HCM was defined as a hypertrophic nondilated LV without other cardiac or systemic diseases capable of producing the magnitude of evident hypertrophy.^1^, ^2^

Specific diagnostic criteria were as follows: (1) maximum LV wall thickness ≥15 mm in ≥1 myocardial segments; or (2) maximum LV wall thickness of 13 to 14 mm, integrating familial context or sporadic occurrence and genotype to allow an informed diagnosis by experienced clinicians. ES‐HCM was defined as ejection fraction <50% with a history of LV hypertrophy compatible with HCM or cellular disarray on endomyocardial biopsy.^20^ Patients with ES‐HCM were excluded from the present study.

Study protocols complied with the Declaration of Helsinki and were approved by the Institutional Review Board of the National Cerebral and Cardiovascular Center (R21042‐9); the execution of the study was also approved at each center. The need to obtain written informed consent was waived because of the retrospective observational and noninvasive nature of the study, and an opt‐out procedure for consent was performed.

### Clinical Evaluation

Baseline clinical data were obtained from medical records within 6 months before or after the enrollment date. Evaluations included blood tests, electrocardiography, transthoracic echocardiography, and ambulatory electrocardiac monitoring, when available.

HCM was classified into 4 subtypes in accordance with previous reports^19^, ^21^: (1) hypertrophic obstructive cardiomyopathy (HOCM) with basal LV outflow tract pressure gradient ≥30 mm Hg at rest or on provocation including exercise; (2) hypertrophic nonobstructive cardiomyopathy with intraventricular pressure gradient <30 mm Hg at rest and on provocation; (3) midventricular obstruction with systolic LV cavity obliteration at the midventricle with peak systolic gradient ≥30 mm Hg; and (4) apical HCM with hypertrophy confined to the LV apex.

Follow‐up information was collected at predefined time points (baseline, 1 year, 5 years, and 10 years). For each participant, observations were administratively censored at the time of the last available follow‐up. Information beyond the last recorded follow‐up was not available, and therefore it was not possible to determine whether participants who were no longer observed had died or were otherwise lost to follow‐up. All clinical outcomes were ascertained through systematic chart review, and ambiguous cases were adjudicated through investigator consensus. Patients lacking any prognostic data were excluded from the analysis.

### Outcome Definitions

The primary clinical end point was cardiovascular death, including SCD, death from heart failure, ventricular arrhythmic events, stroke, and thrombosis. The secondary clinical end points included all‐cause death, hospitalization for heart failure, ventricular arrhythmic events, new‐onset AF, and progression to ES‐HCM. Ventricular arrhythmic events were defined as a composite of SCD, appropriate implantable cardioverter‐defibrillator therapy, or documented sustained ventricular tachycardia or ventricular fibrillation. SCD was defined as instant and unexpected death occurring within the first hour of the onset of symptoms or unwitnessed deaths where the decedent was well 12 to 24 hours before death. New‐onset AF was identified as a first clinical diagnosis confirmed by 12‐lead electrocardiography, ambulatory electrocardiac monitoring, or hospitalization records, and was assessed only in patients without preexisting AF at enrollment. Progression to ES‐HCM was defined by a decline in LV ejection fraction to <50% during the follow‐up period.

### Statistical Analysis

Continuous variables were expressed as mean±SD for normally distributed data or as median with interquartile range (IQR) for nonnormally distributed data. Categorical variables were presented as absolute numbers and percentages. Comparisons between sexes were performed using Student's *t* test for continuous variables and the χ^2^ test for categorical variables. Continuous variables that were not normally distributed were log‐transformed before analysis with the *t* test. Effect sizes were reported as Cohen's *d* for continuous variables and *φ* for categorical variables. Missing values were handled using pairwise deletion. To assess sex differences in medical management, the proportion of patients receiving pharmacological and invasive therapies was compared between the time of enrollment and the latest available follow‐up.

Survival time analyses for each outcome were conducted using the Kaplan–Meier method, with comparisons between groups performed using the log‐rank test. Additionally, univariable and multivariable Cox proportional hazard regression analyses were performed for the primary end point. The proportional hazards assumption was assessed using Schoenfeld residuals. The multivariable model included age, sex, body mass index, family history of sudden death and cardiomyopathy, history of ventricular arrhythmia, history of hypertension, history of AF, advanced New York Heart Association (NYHA) functional class (≥II), morphological phenotypes including obstructive HCM and apical HCM, maximum wall thickness (MWT) adjusted with body surface area (BSA), and medications including β blockers and verapamil/diltiazem. The covariates were determined with reference to previous studies.^7^, ^8^, ^9^, ^11^, ^16^, ^17^, ^18^ Missing data were handled using complete case analysis.

A 2‐sided *P* value <0.05 was considered statistically significant. Statistical analyses were performed using SPSS Statistics version 28.0.0 (IBM, Armonk, NY); EZR version 1.64 (Saitama Medical Center, Jichi Medical University, Saitama, Japan), which is a graphical user interface for R; and R version 4.3.1 (R Foundation for Statistical Computing, Vienna, Austria).

## Results

### Patient Characteristics

Among a total of 3611 patients, 275 with ES‐HCM were excluded; thus, 3336 patients remained. Of these, 3247 patients with available prognostic data were included in the analysis. Baseline characteristics and medical management at initial and latest follow‐up are presented in Tables 1 and 2. Men accounted for the majority of patients with HCM (n=1828; 56%) compared with women, who accounted for 44% (n=1419). The median ages at diagnosis and at enrollment were 65 and 67 years, respectively, and 61% of patients were newly diagnosed with HCM at the time of enrollment. Female patients were significantly older than male patients at both diagnosis (68 [IQR, 58–76] years versus 61 [IQR, 49–70] years; *P*<0.001) and enrollment (70 [IQR, 61–77] years versus 64 [IQR, 54–72] years; *P*<0.001).

**Table 1:** Baseline Characteristics and Events

Variables	Overall	Men	Women	*P* value	Effect size
	n=3247	n=1828	n=1419		
Age at enrollment, y, mean±SD	64±15	61±15	67±15.	<0.001	*d*=15.14
Age at diagnosis, y, mean±SD	61±17	58±16	64±17	<0.001	*d*=16.80
Body mass index, kg/m^2^, mean±SD	23.8±3.9	24.4±3.7	23.0±3.9	<0.001	*d*=3.81
Symptoms, n (%)					
NYHA class ≥II	1376 (42.4)	633 (34.6)	743 (52.4)	<0.001	*φ*=0.18
Chest pain	353 (10.9)	179 (9.8)	174 (12.3)	0.025	*φ*=0.039
Syncope	158 (4.9)	94 (5.2)	64 (4.5)	0.40	
Past history, n (%)					
Atrial fibrillation	741 (22.8)	431 (23.6)	310 (21.8)	0.24	
Ventricular arrhythmia	225 (6.9)	149 (8.2)	76 (5.4)	0.002	*φ*=−0.055
Hypertension	1461 (45.0)	831 (45.5)	630 (44.4)	0.54	
Heart failure hospitalization	200 (6.2)	75 (4.1)	125 (8.8)	<0.001	*φ*=0.097
Family history of HCM, n (%)	338 (10.5)	162 (8.9)	176 (12.6)	<0.001	*φ*=0.059
Family history of SCD, n (%)	265 (8.2)	130 (7.2)	135 (9.6)	0.012	*φ*=0.044
Laboratory data					
BNP, pg/mL, median (IQR)	182.6 (81.4–393.6)	136.8 (64.5–298.4)	255.2 (118.1–496.9)	<0.001	*d*=1.09
N‐terminal pro‐B‐type natriuretic peptide, pg/mL, median (IQR)	832.5 (385.6–1985.3)	617.0 (211.5–1299.0)	1070.0 (560.0–2277.0)	<0.001	*d*=1.16
Echocardiography					
Ejection fraction, %, mean±SD	68±8	67±8	69±8	<0.001	*d*=7.81
LV end‐diastolic diameter, mm, mean±SD	44±6	46±6	42±5	<0.001	*d*=5.58
LV end‐diastolic diameter/BSA, mm/m^2^, mean±SD	27.2±4.0	26.3±3.4	28.4±3.9	<0.001	*d*=3.65
LVDs, mm, mean±SD	26±5	28±5	25±5	<0.001	*d*=4.93
LV systolic diameter/BSA, mm/m^2^, mean±SD	16.3±3.2	15.9±3.0	16.8±3.3	<0.001	*d*=3.13
MWT, mm, mean±SD	16.9±3.8	17.3±4.0	16.5±3.5	<0.001	*d*=3.78
MWT/BSA, mm/m^2^, mean±SD	10.4±2.5	9.9±2.3	11.2±2.5	<0.001	*d*=2.39
Septal E/e′, mean±SD	15.8±7.8	14.2±6.2	18.0±9.1	<0.001	*d*=7.61
Tricuspid regurgitation pressure gradient, mm Hg, mean±SD	26±9	25±8	27±9	<0.001	*d*=8.76
Left atrial volume index, mL/m^2^, mean±SD	50±23	47±22	55±25	<0.001	*d*=23.14
Obstruction >30 mm Hg, n (%)	1029 (31.7)	409 (22.4)	620 (43.7)	<0.001	*φ*=0.23
Max pressure gradient, mm Hg, mean±SD	72±42	63±38	78±43	<0.001	*d*=41.16
Phenotypes of HCM, n (%)					
Hypertrophic nonobstructive cardiomyopathy	1361 (41.9)	849 (46.4)	512 (36.1)	<0.001	*φ*=−0.10
HOCM	828 (25.5)	304 (16.6)	524 (36.9)	<0.001	*φ*=0.23
Midventricular obstruction	201 (6.2)	105 (5.7)	96 (6.8)	0.23	
Apical HCM	857 (26.4)	570 (31.2)	287 (20.2)	<0.001	*φ*=−0.12

BNP and N‐terminal pro‐B‐type natriuretic peptide were log‐transformed for statistical comparison using Welch's *t* test. Effect sizes are reported as Cohen's *d* for continuous variables and *φ* for categorical variables.

BNP indicates brain natriuretic peptide; BSA, body surface area; HCM, hypertrophic cardiomyopathy; HOCM, hypertrophic obstructive cardiomyopathy; IQR, interquartile range; MWT, maximum wall thickness; and NYHA, New York Heart Association.

**Table 2:** Medical Management at Initial and Latest Follow‐Up

Variable	Initial				Latest follow‐up			
	Men	Women	*P* value	Effect size	Men	Women	*P* value	Effect size
Total								
Age, y, mean±SD	61±15	67±15	<0.001	*d*=15.1	67±15	72±15	<0.001	*d*=15.1
Septal reduction therapy, n (%)	28 (1.5)	62 (4.4)	<0.001	*φ*=0.086	121 (6.6)	260 (18.3)	<0.001	*φ*=0.18
Implantable cardioverter‐defibrillator, n (%)	66 (3.6)	42 (3.0)	0.31		215 (11.8)	162 (11.4)	0.76	
AF ablation, n (%)	49 (2.7)	31 (2.2)	0.37		233 (12.7)	164 (11.6)	0.31	
β blocker, n (%)	801 (49.1)	695 (53.2)	0.029	*φ*=0.040	986 (65.1)	872 (73.2)	<0.001	*φ*=0.087
Verapamil/diltiazem, n (%)	114 (7.0)	142 (10.9)	<0.001	*φ*=0.068	109 (7.2)	156 (13.1)	<0.001	*φ*=0.099
Cibenzoline/disopyramide, n (%)	181 (11.1)	274 (21.0)	<0.001	*φ*=0.14	210 (13.9)	328 (27.5)	<0.001	*φ*=0.17
Obstructive HCM								
Age, y, mean±SD	58±16	68±14	<0.001	*d*=14.3	64±15	73±13	<0.001	*d*=14.1
Septal reduction therapy, n (%)	26 (6.4)	53 (8.5)	0.2		106 (25.9)	243 (39.2)	<0.001	*φ*=0.14
Implantable cardioverter‐defibrillator, n (%)	19 (4.6)	20 (3.2)	0.24		54 (13.2)	84 (13.5)	0.87	
AF ablation, n (%)	9 (2.2)	4 (0.6)	0.043	*φ*=0.029	49 (12.0)	57 (9.2)	0.15	
β blocker, n (%)	248 (60.6)	398 (64.2)	0.39		319 (86.9)	479 (87.9)	0.66	
Verapamil/diltiazem, n (%)	32 (7.8)	82 (13.2)	0.022	*φ*=0.086	40 (10.9)	102 (18.7)	0.001	*φ*=0.11
Cibenzoline/disopyramide, n (%)	109 (27.7)	210 (33.9)	0.037	*φ*=0.080	139 (37.9)	271 (49.7)	<0.001	*φ*=0.12
Hypertrophic nonobstructive cardiomyopathy								
Age, y, mean±SD	61±16	63±18	<0.001	*d*=16.7	67±16	68±18	0.63	
Implantable cardioverter‐defibrillator, n (%)	43 (5.1)	20 (3.9)	0.32		111 (13.1)	53 (10.4)	0.14	
AF ablation, n (%)	24 (2.8)	17 (3.3)	0.61		107 (12.6)	69 (13.5)	0.64	
β blocker, n (%)	373 (49.6)	209 (43.9)	0.052		448 (62.6)	276 (62.4)	0.97	
Verapamil/diltiazem, n (%)	50 (5.9)	41 (8.0)	0.013	*φ*=0.080	42 (5.9)	33 (7.5)	0.28	
Cibenzoline/disopyramide, n (%)	52 (6.1)	52 (10.2)	0.001	*φ*=0.098	52 (7.3)	50 (11.3)	0.018	*φ*=0.069
Apical HCM								
Age, y, mean±SD	64±13	72±11	0.003	*d*=12.2	70±13	76±10	<0.001	*d*=12.0
Implantable cardioverter‐defibrillator, n (%)	4 (0.7)	2 (0.7)	0.99		50 (8.8)	25 (8.7)	0.98	
AF ablation, n (%)	16 (2.8)	10 (3.5)	0.59		77 (13.5)	38 (13.2)	0.91	
β blocker, n (%)	180 (31.6)	88 (30.7)	0.78		219 (50.7)	117 (57.4)	0.12	
Verapamil/diltiazem, n (%)	32 (5.6)	19 (6.6)	0.63		27 (6.3)	21 (10.3)	0.07	
Cibenzoline/disopyramide, n (%)	20 (3.5)	12 (4.2)	0.67		19 (4.4)	7 (3.4)	0.57	

Obstructive HCM included hypertrophic obstructive cardiomyopathy and midventricular obstruction. Effect sizes are reported as Cohen's *d* for continuous variables and *φ* for categorical variables.

AF indicates atrial fibrillation; HCM, hypertrophic cardiomyopathy.

At enrollment, female patients were more likely to be symptomatic with NYHA functional classes II to IV compared with male patients (*P*<0.001). They also had a greater history of heart failure, higher serum BNP (brain natriuretic peptide) levels, and echocardiographic findings suggestive of diastolic dysfunction, including elevated septal E/e' and increased left atrial volume index. However, no sex differences were observed in the history of AF.

### Outcome

**Figure 1:**
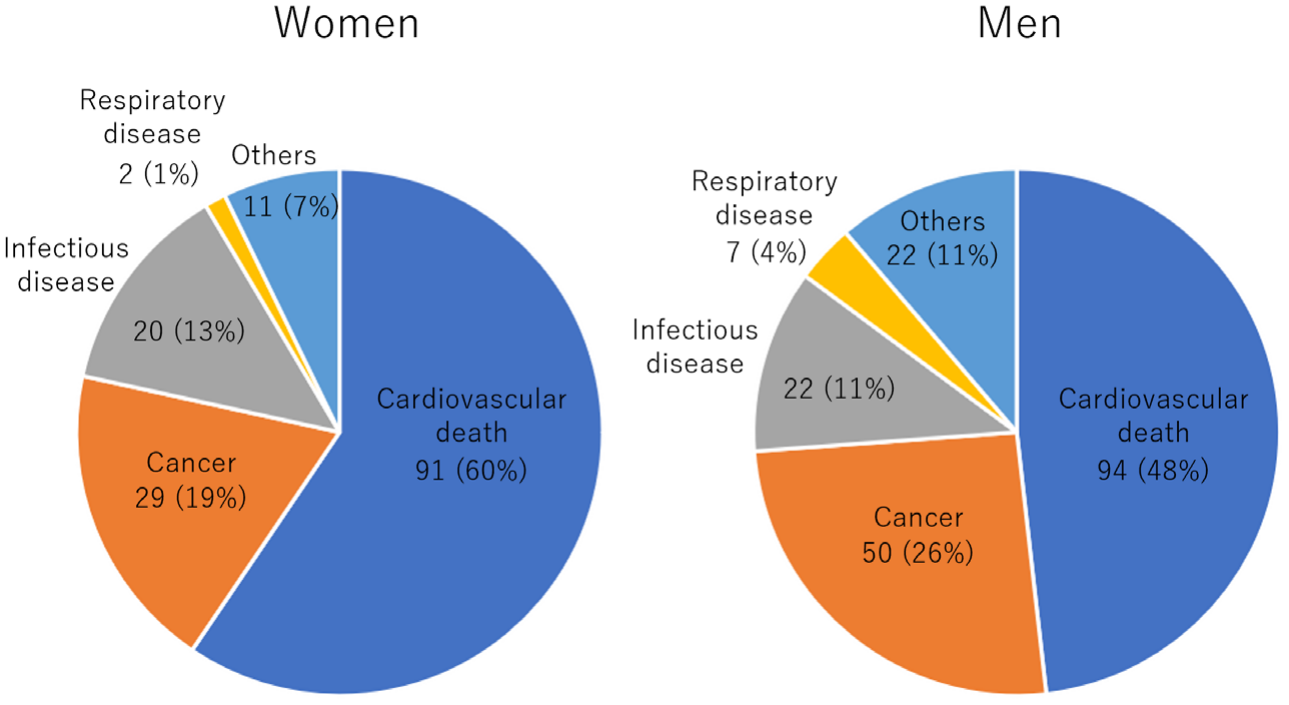
Cause‐specific composition of all‐cause deaths in male and female patients with hypertrophic cardiomyopathy. Each segment represents the number of deaths and the percentage of the total deaths in each sex category.

**Figure 2:**
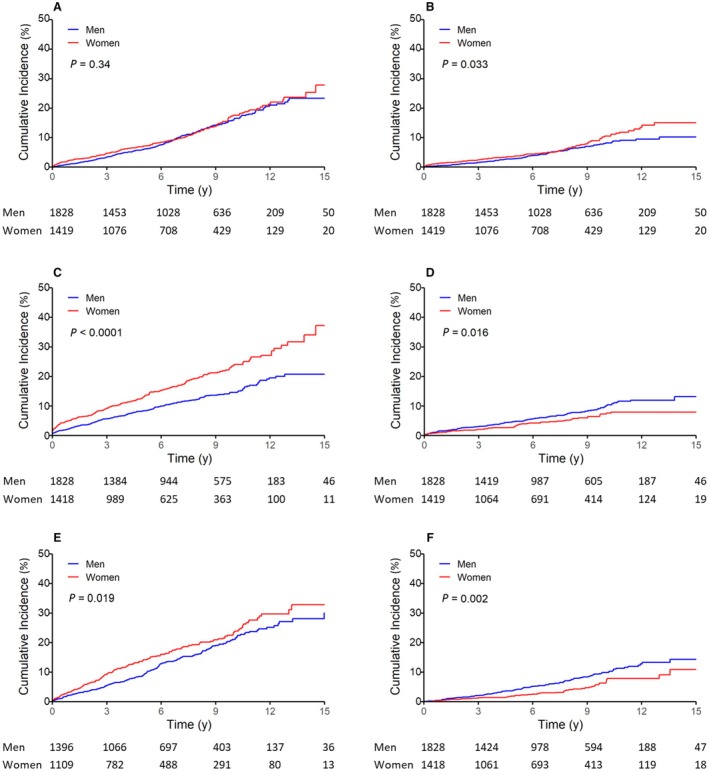
Comparison of cumulative incidence of each event in men and women with hypertrophic cardiomyopathy with Kaplan–Meier curves and log‐rank test. (**A**), All‐cause death; (**B**) cardiovascular death; (**C**) heart failure hospitalization; (**D**) ventricular arrhythmic events; (**E**) new‐onset atrial fibrillation; (**F**) progression to end‐stage hypertrophic cardiomyopathy.

The median follow‐up duration was longer among male patients (6.8 (3.6–10.1) versus 5.9 (3.1–9.7) years; *P*<0.001). During the follow‐up period, a total of 369 patients (11.4%) died, of which 184 patients (5.7%) were cardiovascular deaths. The cause‐specific composition of all‐cause deaths is illustrated in Figure 1. Kaplan–Meier analysis demonstrated that no significant difference was observed in all‐cause death, whereas women exhibited a higher risk of cardiovascular death (log‐rank test; *P*=0.34 and *P*=0.033, respectively; Figure 2A and 2B). Heart failure hospitalizations and new‐onset AF occurred more frequently in women (log‐rank test; *P*<0.001 and *P*=0.019, respectively; Figure 2C and 2E). In contrast, men were more likely to experience ventricular arrhythmic events and progression to the end stage (log‐rank test; *P*=0.016 and *P*=0.002, respectively; Figure 2D and 2F).

### Evaluation of Prognostic Factors in Female Patients

**Figure 3:**
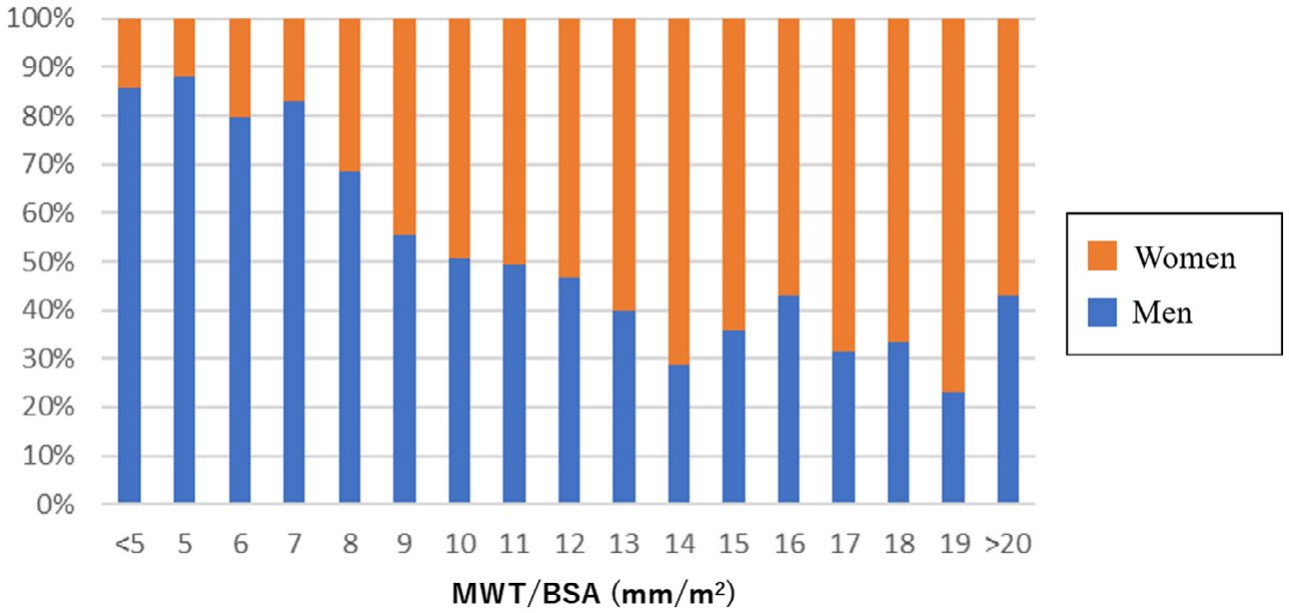
Sex ratio by MWT/BSA. BSA indicates body surface area; and MWT, maximal wall thickness.

Echocardiography revealed sex‐specific morphological characteristics: Female patients exhibited higher LV ejection fraction and smaller LV diastolic dimensions and MWT. However, after adjusting LV end‐diastolic diameter and MWT for BSA, female patients were found to have significantly larger LV end‐diastolic diameter and greater wall thickness. As shown in Figure 3, the proportion of male patients was markedly higher in the lower MWT/BSA group, whereas in the higher MWT/BSA group, the sex ratio was comparable or even higher among women. This inverse distribution pattern suggests that after accounting for body size, women tended to have relatively greater wall thickness compared with men.

**Figure 4:**
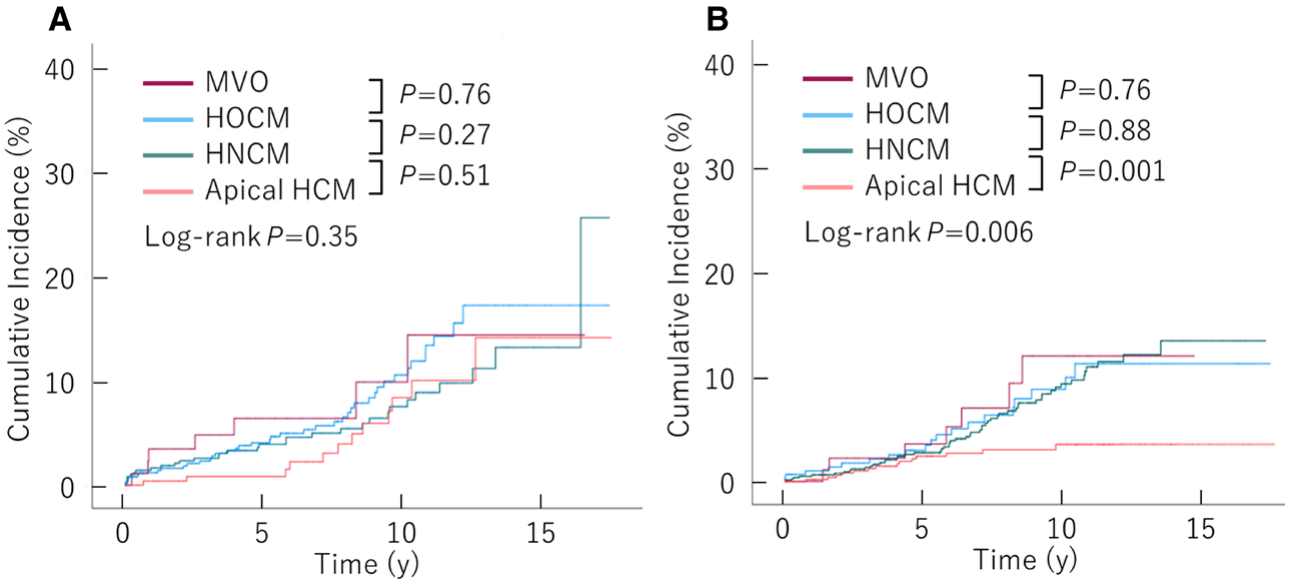
Cumulative incidence of cardiovascular death among morphological subtypes of HCM in women and men. Comparison of cumulative incidence of each phenotype with Kaplan–Meier curves and log‐rank test. **A**, Survival time analysis in women; (**B**) survival time analysis in men. HCM indicates hypertrophic cardiomyopathy; HNCM, hypertrophic nonobstructive cardiomyopathy; HOCM, hypertrophic obstructive cardiomyopathy; and MVO, midventricular obstruction.

LV outflow tract obstruction was more common in female patients and was present in 37% of cases, whereas apical HCM was significantly more prevalent among male patients. Midventricular obstruction was observed in only 6.2% of patients, with no significant sex differences. When cardiovascular death was analyzed according to HCM phenotypes, apical HCM was associated with a significantly better prognosis in men. In women, all 4 phenotypes (HOCM, hypertrophic nonobstructive cardiomyopathy, midventricular obstruction, and apical HCM) exhibited similar prognoses with no significant differences (Figure 4).

In the overall HCM cohort, β blockers, nondihydropyridine calcium channel blockers, and class I antiarrhythmic drugs (AADs) were prescribed more frequently, and septal reduction therapy was performed more often in women (Table 2). Similar trends were observed in obstructive HCM. In patients with hypertrophic nonobstructive cardiomyopathy, baseline use of nondihydropyridine calcium channel blockers and class I AADs was more common in women; however, the difference in nondihydropyridine calcium channel blocker use had disappeared at the latest follow‐up. No treatment differences were observed between men and women in apical HCM (Table 2).

In univariable analysis, age, NYHA class ≥II, MWT/BSA, and the absence of septal reduction therapy in patients with obstructive HCM was significantly associated with poor prognosis, while apical HCM was related to favorable prognosis (Table 3). In the multivariable analysis model 2, age, NYHA class ≥II, MWT/BSA, and apical HCM were independently associated with prognosis. Consequently, the hazard ratio (HR) for cardiovascular death in women decreased, and the sex difference was no longer observed (HR, 1.041 [95% CI, 0.767–1.413]; and HR, 0.738 [95% CI, 0.511–1.067]; *P*=0.80 and *P*=0.11, respectively; Table 3). The proportional hazards assumption was assessed using Schoenfeld residuals, and no significant violation was observed for the overall model (global test, *P*=0.287). The proportion of missing data in the multivariable analysis was 21.2%, with a particularly high rate of missingness for class IA AAD use (Table S1). Compared with the analyzed cohort, patients with missing data were older and less likely to be symptomatic. Morphologically, this group included a higher proportion of patients with apical hypertrophic cardiomyopathy (Table S2).

**Table 3:** Univariable and Multivariable Models Predicting Cardiovascular Death

Variables	Univariate		Multivariate	
	HR (95% CI)	*P* value	HR (95% CI)	*P* value
Female sex	1.366 (1.024–1.823)	0.034	0.738 (0.511–1.067)	0.11
Age, y	1.055 (1.040–1.070)	<0.001	1.039 (1.023–1.056)	<0.001
Body mass index, kg/m^2^	0.929 (0.889–0.969)	<0.001	0.969 (0.923–1.018)	0.21
Family history of SCD	0.599 (0.325–1.102)	0.1	0.711 (0.376–1.344)	0.29
Family history of HCM	0.619 (0.359–1.069)	0.085	0.906 (0.480–1.708)	0.76
Hypertension	1.242 (0.930–1.659)	0.14	1.083 (0.781–1.502)	0.63
Ventricular arrhythmia	1.231 (0.747–2.028)	0.42	1.144 (0.662–1.976)	0.63
Atrial fibrillation	2.269 (1.685–3.055)	<0.001	1.548 (1.101–2.177)	0.012
NYHA class ≥II	2.369 (1.759–3.192)	<0.001	1.986 (1.399–2.819)	<0.001
MWT/BSA, mm/m^2^	1.145 (1.092–1.200)	<0.001	1.124 (1.057–1.195)	<0.001
Apical HCM	0.462 (0.305–0.699)	<0.001	0.533 (0.324–0.876)	0.013
Obstructive HCM with septal reduction therapy	0.892 (0.561–1.419)	0.63	0.643 (0.355–1.163)	0.14
Obstructive HCM without septal reduction therapy	1.797 (1.307–2.471)	<0.001	1.172 (0.768–1.787)	0.46
β blocker use	0.883 (0.649–1.200)	0.43	0.768 (0.538–1.095)	0.15
Verapamil/Diltiazem use	1.483 (0.979–2.245)	0.063	1.461 (0.917–2.328)	0.11
Cibenzoline/Disopyramide use	1.087 (0.749–1.579)	0.66	0.930 (0.601–1.440)	0.75

Obstructive HCM included hypertrophic obstructive cardiomyopathy and midventricular obstruction.

BSA indicates body surface area; HCM, hypertrophic cardiomyopathy; HR, hazard ratio; MWT, maximum wall thickness; NYHA, New York Heart Association; and SCD, sudden cardiac death.

## Discussion

The present study was a large, retrospective, observational analysis investigating sex differences in a Japanese cohort of patients with HCM. The major findings were as follows: (1) Women demonstrated a higher risk of cardiovascular death compared with men; (2) women were older, more symptomatic, and more frequently hospitalized for heart failure and newly diagnosed with AF during follow‐up; and (3) age, NYHA class ≥II, history of atrial fibrillation, MWT/BSA, and apical HCM were associated with prognosis. After adjustment for these factors in the multivariable model, the sex difference in cardiovascular death was no longer statistically significant.

### Patient Characteristics

In line with previous epidemiological data, the majority of HCM patients were men. Prior Japanese studies reported male predominance rates of 67% to 72%, with similar observations in Chinese, Korean, and Western cohorts.^7^, ^8^, ^10^, ^12^, ^13^, ^14^, ^16^, ^22^, ^23^, ^24^ Although the precise reasons for this sex imbalance remain unclear, differences in genetic penetrance and modifiers have been proposed. For example, *MYBPC3* variant carriers demonstrate delayed penetrance in women compared with men, whereas *MYH7* mutations appear to exert similar effects across sexes.^11^ As discussed later, uniform diagnostic criteria are associated with underdiagnosis in women, which may explain male predominance and delayed penetrance in *MYBPC3* carriers.

Patients in this study were notably older at diagnosis compared with those reported in Western studies, where mean ages often fall in the fifth decade. Prior Japanese studies have similarly documented higher mean ages in the 60s. A plausible explanation is the high prevalence of apical HCM in Asian populations, reported to account for 15% to 25% of cases, which tends to present later due to its relatively benign early clinical course.^25^, ^26^ Despite the lower proportion of female patients, women presented at an older age and with more pronounced symptoms. This trend is similar in most previous studies, indicating that it is an important characteristic of female patients.^7^, ^8^, ^9^, ^11^, ^12^

Our findings demonstrated that women were at higher risk of cardiovascular death, aligning with prior studies, including a meta‐analysis by Zhao et al.^27^ In contrast, all‐cause death did not differ by sex in Kaplan–Meier analysis. The excess all‐cause death in men was largely attributable to noncardiovascular causes such as malignancy and infection, which accounted for 52% of all‐cause deaths. This likely reflected the older age of our study population compared with previous studies.^7^, ^13^ These findings suggest that in older Japanese populations, cardiovascular death may serve as a more optimal end point for evaluating sex‐based prognostic differences in HCM, as it more directly reflects disease‐related risk.

### Possible Determinants of Poor Prognosis in Female Patients With HCM

**Figure 5:**
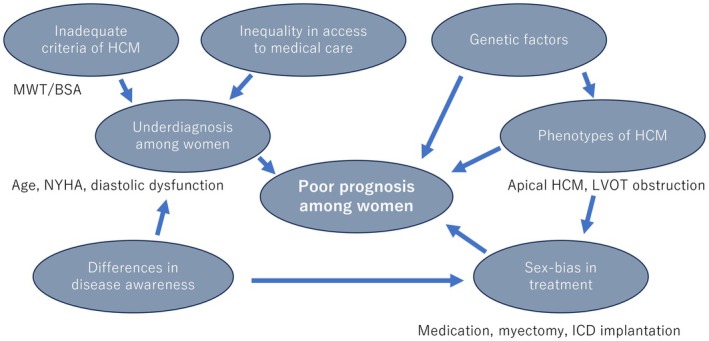
Possible determinants of poor prognosis in female patients with HCM. BSA indicates body surface area; HCM, hypertrophic cardiomyopathy; ICD, implantable cardioverter‐defibrillator; LVOT, left ventricular outflow tract; MWT, maximal wall thickness; and NYHA, New York Heart Association.

Sex differences in HCM are influenced by multiple factors, including genetic background, social environment, awareness of disease, and potential biases in diagnosis and medical management (Figure 5).^7^, ^9^, ^16^, ^17^, ^18^, ^28^ Because the relative contribution of these factors may vary across races and ethnicities, it is important to identify which factors are most relevant in each patient population.

### Inadequate Diagnostic Criteria of HCM

The sex‐neutral cutoff value of 15 mm for LV wall thickness may partly account for the observed sex differences. This is because female patients exhibited smaller LV dimensions and wall thicknesses than males, with upper normal limits for wall thickness approximately ≈1 mm lower in women, as consistently reported in both Japanese and Western reference populations.^29^, ^30^ To correct for this potential bias, previous reports have recommended adjusting wall thickness for age and body size; therefore, in the present study, we normalized wall thickness using BSA.^17^

Before adjustment, women had significantly smaller wall thickness than men; however, after indexing to BSA, women demonstrated greater wall thickness. Moreover, the proportion of male patients was higher in those with MWT/BSA <10, while the sex ratio became more balanced as MWT/BSA increased. This finding suggests that women with relatively mild hypertrophy may be underdiagnosed. Conversely, at higher MWT/BSA levels, the proportion of women exceeded that of men, indicating that women were more likely to have advanced hypertrophy at the baseline.

Because greater wall thickness has been associated with poor outcomes, and MWT/BSA was independently correlated with adverse prognosis in our multivariable analysis, the bias introduced by inappropriate diagnostic thresholds may represent 1 of the underlying mechanisms contributing to the observed sex differences. Rather than applying uniform diagnostic criteria, consideration should be given to individually tailored approaches, such as MWT adjusted with BSA or the criteria proposed by Shiwani et al.^17^

### Underdiagnosis Among Women

In this cohort, women were ≈6 years older than men at enrollment and reported more severe symptoms at diagnosis, findings that align with prior studies.^7^, ^8^, ^9^, ^11^, ^12^ These data highlight ongoing concerns about potential underdiagnosis of HCM in women. Age at presentation and NYHA class ≥ II were strongly associated with prognosis in the multivariable analysis, suggesting that underdiagnosis in women may have contributed to the observed sex differences in outcomes.

Several contributing factors may underlie these findings, such as inadequate criteria of HCM and inequality in access to medical care. Disparities in health care access in Japan are prominent. According to the 2019 Summary Report of the Comprehensive Survey of Living Conditions by the Ministry of Health, Labour and Welfare, the participation rate of women in routine health checkups was 65.6%, lower than that of men at 74%, as shown in Table 15 of the report.^31^ Housewives had been reported to have a particularly low rate of health checkups, which may contribute to the disproportionate access between sexes.^32^ Limited screening opportunities could lead to delayed recognition of asymptomatic or early‐stage disease.

This underdiagnosis in women may also imply a longer period of unrecognized and untreated disease, which could theoretically contribute to more advanced myocardial fibrosis. Indeed, some studies have reported higher degrees of myocardial fibrosis on cardiac MRI in women with HCM.^33^ However, the evidence on sex differences in myocardial fibrosis in HCM remains inconsistent.^34^ Thus, whether a longer period of untreated disease causally contributes to greater myocardial fibrosis and, consequently, poorer outcomes in women remains speculative and warrants further investigation.

### Differences of Morphological Phenotypes Between Sexes

It is well established that the prognosis of HCM varies depending on its morphological phenotype.^35^, ^36^ In our cohort, distinct sex‐related distributions of these phenotypes were observed: apical HCM, which was associated with a relatively favorable prognosis, was more common in men, whereas HOCM, linked to poor prognosis, was more frequent in women. However, Kaplan–Meier analysis showed that prognosis was equivalent for all conditions except male apical HCM, suggesting that the higher prevalence of apical HCM among men could partly account for their better overall prognosis in this cohort.

No reports have clearly demonstrated a sex difference in prognosis for apical HCM. Interestingly, this study indicates that apical HCM in women does not necessarily have a favorable prognosis, which may also contribute to the poor prognosis observed in women. Although Table 2 indicates no significant differences in treatment between men and women, women with apical HCM constituted the oldest subgroup, a factor that may be associated with adverse prognosis. Further investigation is needed on sex differences in the prognosis of apical HCM.

### Sex Bias in Medical Management

According to the Japanese Circulation Society,^21^ American Heart Association,^28^ and European Society of Cardiology guidelines,^1^ β blockers, nondihydropyridine calcium channel blockers, cibenzoline/disopyramide, and septal reduction therapy are standard treatments for HCM. While these should be administered equally to men and women, disparities exist in practice. For example, reports indicate that β blockers are more frequently prescribed to men, while nondihydropyridine calcium channel blockers are more commonly prescribed to women.^16^, ^37^ Given that therapeutic management may substantially affect prognosis, we examined potential sex differences in treatment patterns.

When comparing treatment rates at baseline and at the latest follow‐up, women with obstructive HCM tended to receive more active treatment. A previous Swedish study reported suboptimal β blocker use in women with HOCM, which was associated with worse prognosis; however, in the present Japanese cohort, there was no sex difference in β blocker use, and septal reduction therapy, nondihydropyridine calcium channel blockers, and class I AADs were all more frequently used in women than in men.^16^ The prevalence of hypertension did not differ between sexes in this cohort, suggesting that this difference is likely to reflect their use as HCM‐directed therapy. Conversely, among patients with hypertrophic nonobstructive cardiomyopathy, nondihydropyridine calcium channel blockers and class I AADs were also more frequently used in women. Although this suggested that patients whose pressure gradient resolved with treatment for HOCM may have been included, this could not be verified in this study due to insufficient prebaseline information.

Collectively, these findings suggest that, in Japanese patients with HCM, women tend to receive adequate or even more intensive treatment compared with men. Importantly, multivariate analysis revealed no significant association between these treatments and adverse outcomes. Therefore, differences in medical management are unlikely to fully account for the poorer prognosis observed in women.

Nevertheless, caution is warranted. The absence of detailed information on oral medication dosages in this study may have led to an underestimation of their potential impact on prognosis. In addition, although calcium channel blocker use was not independently associated with outcome in multivariable analysis, a trend toward association was observed. Thus, a potential relationship between calcium channel blocker therapy and adverse outcomes cannot be completely excluded. Given the limited information on treatment intensity and the possibility of a type II error, the prognostic impact of specific pharmacological therapies warrants further investigation specifically designed to address this question.

### What Factors Shape Sex Differences Among Japanese Patients With HCM?

As summarized above, our analyses identified several factors that contributed to sex differences in prognosis among Japanese patients with HCM. Inappropriate diagnostic thresholds, underdiagnosis in women, and differences in morphological phenotypes were each associated with poor prognosis in female patients. In contrast, despite prior reports suggesting that suboptimal disease management may worsen prognosis in women, women in our cohort appeared to receive adequate therapy, making therapeutic bias an unlikely explanation. AF was also significantly associated with cardiovascular death; however, it does not account for sex differences because its prevalence did not differ between men and women. After adjustment for these factors in the multivariable analysis, the sex difference in cardiovascular death disappeared, suggesting that the poor prognosis observed in Japanese women with HCM can largely be attributed to these factors.

## Study Limitations

Several limitations of this study warrant consideration.

First, as a retrospective observational study, causal relationships could not be definitively established, and potential unmeasured confounding factors may have influenced the observed associations. Moreover, the absence of genetic testing precluded the assessment of genotype–sex interactions. Although prior studies suggest that women harbor a higher prevalence of pathogenic mutations, adjustment for genotype did not fully explain sex‐based differences in clinical outcomes,^11^ underscoring the need for future prospective studies incorporating comprehensive genetic analyses.

In addition, this study was characterized by a relatively high proportion of missing data in the multivariable analysis. A relatively large proportion of patients with missing data had apical HCM, which may have contributed to the observed characteristics of older age and fewer symptoms. Furthermore, differences in medication use, particularly in β blockers and class IA AADs, may have introduced bias.

Second, event rates were analyzed from the time of enrollment onward. Patients who died suddenly before a definitive HCM diagnosis were not captured, potentially leading to an underestimation of the true incidence of SCD and arrhythmic events.

Third, baseline differences in disease severity between men and women likely reflect not only biological factors but also social determinants such as disparities in health care access. This heterogeneity in enrollment profiles may have contributed to the observed sex differences in outcomes, a challenge similarly faced by prior cohort studies.^7^, ^8^, ^11^, ^12^, ^13^

Despite these limitations, the study provides important insights into sex‐related differences in the clinical course and outcomes of HCM in a large Japanese cohort.

## Conclusions

A retrospective observational study in Japan revealed sex‐related differences in clinical characteristics and outcomes in HCM. Although women tended to receive adequate medical management, women exhibited higher rates of cardiovascular death. In Japanese patients with HCM, inappropriate diagnostic thresholds, delayed diagnosis, and differences in morphological phenotypes appeared to contribute to the observed sex differences.

## Sources of Funding

This work was supported by Japan Agency for Medical Research and Development (Grant No. JP23ek0109539).

## Disclosures

Dr Abe has received personal fees from Boehringer Ingelheim Japan. Dr Ueda has received research grants from Abbott, Daiichi‐Sankyo, Teijin, Japan Lifeline, Orbus Neich, Janssen, Otsuka, Ono, Eli Lilly, Amgen, Novo Nordisk, and Novartis; and has received lecture fees from Abbott, Kowa, Bayer, Daiichi‐Sankyo, Nipro, Takeda, AstraZeneca, Japan Lifeline, Novartis, Ono, Boehringer Ingelheim, PDR Pharma, Kaneka Medix, Mochida, Otsuka, and Amgen. Dr Sakata has received grants or honoraria from Abbott Medical Japan, Otsuka Pharmaceutical, Nippon Boehringer Ingelheim, AstraZeneca, Novartis Pharma, and Bayer. Dr Furukawa has received personal fees from Novartis. Dr Ando has received personal fees from Abbott Medical Japan, Medtronic Japan, and Biotronik. Dr Iwakura has received speaker honoraria from AstraZeneca and Eli Lilly and Company. Dr Kitamura has received personal fees from Daiichi‐Sankyo and Abbott Medical Japan. Dr Izumi has received speaker honoraria from Daiichi‐Sankyo, Nippon Boehringer Ingelheim, and Novartis; and has received research funding from Pfizer, LSI Medience, PPD‐SNBL, Abbott Medical Japan, Bristol Myers Squibb, and Eli Lilly and Company. The remaining authors have no disclosures to report.

## Supporting information

Tables S1–S2
REVEAL‐HCM Investigators Group

STROBE checklist
