# Supplementary material for: Sex Differences in Characteristics and Outcomes Among Patients With Hypertrophic Cardiomyopathy: Insights From the REVEAL‐HCM Study
Source: J Am Heart Assoc. 2026 Jun 23;15(13):e046708. doi: 10.1161/JAHA.125.046708 (PMC13477277; doi:10.1161/JAHA.125.046708)
Supplement: Supplementary file 2 [file JAH3-15-e046708-s002.pdf]

SUPPLEMENTAL MATERIAL

APPENDIX

The REVEAL-HCM Investigators Group:

Toru Kubo, MD, PhD, *Department of Cardiology and Geriatrics, Kochi Medical School, Kochi University, Kochi, Japan.*

Kenta Sugiura, MD, PhD, *Department of Cardiology and Geriatrics, Kochi Medical School, Kochi University, Kochi, Japan.*

Rika Taneike, MD, PhD, *Department of Cardiovascular Medicine, Osaka University Graduate School of Medicine, Osaka, Japan.*

Yasuharu Takeda, MD, PhD, *Department of Cardiovascular Medicine, Osaka University Graduate School of Medicine, Osaka, Japan.*

Keishi Moriwaki, MD, PhD, *Department of Cardiology and Nephrology, Mie University Graduate School of Medicine.*

Ryuji Okamoto, MD, PhD, *Department of Cardiology and Nephrology, Mie University Graduate School of Medicine.*

Hitoshi Takano, MD, PhD, *Department of Cardiovascular Medicine, Nippon Medical School, Tokyo, Japan.*

Yoichi Imori, MD, PhD, *Department of Cardiovascular Medicine, Nippon Medical School, Tokyo, Japan.*

Hiroki Shiomi, MD, PhD, *Department of Cardiovascular Medicine, Faculty of Medicine, Kyoto University Graduate School of Medicine, Kyoto, Japan.*

Yuki Obayashi, MD, PhD, *Department of Cardiovascular Medicine, Faculty of Medicine, Kyoto University Graduate School of Medicine, Kyoto, Japan.*

Taiji Okada, MD, PhD, *Department of Cardiovascular Medicine, Kobe City Medical Center General Hospital.*

Ryo Shigeno, MD, PhD, *Department of Cardiovascular Medicine, Kobe City Medical Center General Hospital.*

Toru Hashimoto, MD, PhD, *Department of Cardiovascular Medicine, Faculty of Medical Sciences, Kyushu University*.

Shintaro Kinugwa, MD, PhD, *Department of Cardiovascular Medicine, Faculty of Medical Sciences, Kyushu University*.

Yodo Tamaki, MD, PhD, *Department of Cardiology, Tenri Hospital, Tenri, Japan.*

Yukihiro Hamaguchi, MD, PhD, *Department of Cardiovascular Medicine, Shizuoka General Hospital.*

Kenichi Ishizu, MD, PhD, *Department of Cardiology, Kokura Memorial Hospital, Kitakyushu, Japan.*

Mariko Kitano, MD, PhD, *Cardiovascular Center, Medical Research Institute Kitano Hospital, Osaka, Japan.*

Toka Hamaguchi, MD, PhD, *Cardiovascular Center, Medical Research Institute Kitano Hospital, Osaka, Japan.*

Sachiyo Ono, MD, *Department of Cardiovascular Medicine, Kurashiki Central Hospital, Kurashiki, Japan.*

Yoshiki Matsumura, MD, PhD, *Department of Cardiology, Osaka City General Hospital, Osaka, Japan.*

Kiyotaka Watanabe, MD. PhD, *Department of Cardiology, Suzuka General Hospital, Suzuka, Japan.*

Yuichi Sato, MD, *Department of Cardiology, Matsusaka Chuo General Hospital, Matsusaka, Japan.*

Takafumi Koji, MD, *Department of Cardiology, Owase General Hospital, Owase, Japan.*

Soichiro Kobayashi, MD, *Department of Cardiology, Ise Red Cross Hospital, Ise, Japan.*

Table S1. Missing rate for each covariate

Covariates	Missing rate, n (%)
Female gender	0 (0)
Age (y)	0 (0)
BMI (kg/m2)	172 (5.3)
Family history of SCD	33 (1.0)
Family history of HCM	34 (1.0)
Hypertension	1 (0)
Ventricular arrhythmia	0 (0)
Atrial fibrillation	0 (0)
NYHA≥II	0 (0)
MWT/BSA (mm/m2)	211 (6.5)
Apical HCM	0 (0)
Obstructive HCM with SRT	0 (0)
Obstructive HCM without SRT	0 (0)
Beta blocker use	125 (3.8)
Verapamil/Diltiazem use	125 (3.8)
Cibenzoline/Disopyramide use	502 (15.5)

BMI, body mass index; SCD, sudden cardiac death; HCM, hypertrophic cardiomyopathy; NYHA, New York Heart Association; MWT, maximum wall thickness; BSA, body surface area; SRT, septal reduction therapy.

Table S2. Comparison of patient characteristics between the missing case and complete case groups

Variables	complete case n=2559	missing case n=688	*p* value
Age at enrollment, mean±SD, y	63 ± 15	66 ± 15	<0.001
Age at diagnosis, mean±SD, y	60 ± 17	64 ± 17	<0.001
Female	1136 (44)	283 (41)	0.13
BMI, mean±SD, kg/m^2	23.8 ± 3.9	23.6 ±3.7	0.064
Symptoms, n (%)			
NYHA≧II	1184 (46)	192 (28)	<0.001
Chest pain	300 (12)	53(8)	0.003
Syncope	136 (5)	22 (3)	0.022
Past history, n (%)			
Atrial fibrillation	597 (23)	144 (21)	0.18
Ventricular arrhythmia	198 (8)	27 (4)	<0.001
Hypertension	1174 (46)	287 (42)	0.055
Heart failure hospitalization	167 (7)	33 (5)	0.094
Family history of HCM, n (%)	299 (12)	39 (6)	<0.001
Family history of SCD, n (%)	235 (9)	30 (5)	<0.001
Laboratory data			
BNP, median (IQR), pg/mL	188 (104-465)	129 (76-551)	0.19
NT-proBNP, median (IQR), pg/mL	1018 (400-2877)	998 (530-3760)	0.35
Echocardiography			
EF, mean±SD, %	68 ± 8	68 ± 8	0.39
LVDd, mean±SD, mm	44 ± 6	44 ± 6	0.23
LVDd/BSA, mean±SD,mm/m^2	27.1 ± 3.8	27.5 ± 3.9	0.04
LVDs, mean±SD, mm	26 ± 5	26 ± 5	0.67
LVDs/BSA, mean±SD, mm/m^2	16.2 ± 3.2	16.5 ± 3.1	0.073
MWT, mean±SD, mm	17.1 ± 3.9	16.3 ± 3.4	<0.001
MWT/BSA, mean±SD, mm/m^2	10.5 ± 2.5	10.0 ± 2.2	<0.001
septal E/e', mean±SD	16.0 ± 7.9	15.2 ± 7.6	0.035
TRPG, mean±SD, mmHg	26 ± 9	25 ± 9	0.013
LAVI, mean±SD, ml/m^2	51 ± 24	49 ± 23	0.27
Obstruction >30mmHg, n (%)	888 (35)	141 (21)	<0.001
Max PG, mean±SD, mmHg	72 ± 42	69 ± 41	0.39
Phenotypes of HCM, n (%)			

HNOCM	1101 (43)	260 (38)	0.014
LVOTO	724 (28)	104 (15)	<0.001
MVO	164 (6)	37 (5)	0.32
apical HCM	570 (22)	287 (42)	<0.001
Medical management			
Septal reduction therapy, n (%)	72 (3)	18 (3)	0.78
ICD, n (%)	90 (4)	18 (3)	0.24
AF ablation, n (%)	61 (2)	19 (3)	0.57
Beta blocker, n (%)	1274 (52)	222 (44)	<0.001
verapamil/diltiazem, n (%)	217 (9)	39 (8)	0.36
Cibenzoline/Disopyramide, n (%)	418 (17)	37 (7)	<0.001

BNP and NT-proBNP were log-transformed for statistical comparison using Welch's t-test. BMI, body mass index; BNP, brain natriuretic peptide; BSA, body surface area; EF, ejection fraction; eGFR, estimated glomerular filtration rate; HCM, hypertrophic cardiomyopathy; HNCM, hypertrophic non-obstructive cardiomyopathy; HOCM, hypertrophic obstructive cardiomyopathy; ICD, Implantable Cardioverter Defibrillator; IQR, interquartile range; LAVI, left atrial volume index; LVDd, left ventricular diastolic diameter; MVO, mid ventricular obstruction; MWT, maximum wall thickness; NT-proBNP, N-terminal pro brain natriuretic peptide; NYHA, New York Heart Association; PG, pressure gradient; SCD, sudden cardiac death; SD, standard division; TRPG, tricuspid regurgitation pressure gradient.
